# Key Players of Cisplatin Resistance: Towards a Systems Pharmacology Approach

**DOI:** 10.3390/ijms19030767

**Published:** 2018-03-07

**Authors:** Navin Sarin, Florian Engel, Florian Rothweiler, Jindrich Cinatl, Martin Michaelis, Roland Frötschl, Holger Fröhlich, Ganna V. Kalayda

**Affiliations:** 1 Institute of Pharmacy, Clinical Pharmacy, University of Bonn, 53121 Bonn, Germany; n.sarin@uni-bonn.de; 2 Federal Institute for Drugs and Medical Devices (BfArM), 53175 Bonn, Germany; engel_florian@hotmail.de (F.E.); Roland.Froetschl@bfarm.de (R.F.); 3 Institute of Medical Virology, Goethe University Hospital Frankfurt, 60596 Frankfurt/Main, Germany; f.rothweiler@kinderkrebsstiftung-frankfurt.de (F.R.); cinatl@em.uni-frankfurt.de (J.C.J.); 4 Industrial Biotechnology Centre and School of Biosciences, School of Biosciences, University of Kent, Canterbury CT2 7NJ, UK; M.Michaelis@kent.ac.uk; 5 Bonn-Aachen International Center for IT (b-it), Life Science Data Analytics & Algorithmic Bioinformatics, University of Bonn, 53115 Bonn, Germany; frohlich@bit.uni-bonn.de

**Keywords:** cisplatin resistance, cellular signalling, HRas, p38, CCL2, DOK1, PTK2B, JNK3

## Abstract

The major obstacle in the clinical use of the antitumor drug cisplatin is inherent and acquired resistance. Typically, cisplatin resistance is not restricted to a single mechanism demanding for a systems pharmacology approach to understand a whole cell’s reaction to the drug. In this study, the cellular transcriptome of untreated and cisplatin-treated A549 non-small cell lung cancer cells and their cisplatin-resistant sub-line A549^r^CDDP^2000^ was screened with a whole genome array for relevant gene candidates. By combining statistical methods with available gene annotations and without a previously defined hypothesis *HRas, MAPK14* (p38)*, CCL2, DOK1* and *PTK2B* were identified as genes possibly relevant for cisplatin resistance. These and related genes were further validated on transcriptome (qRT-PCR) and proteome (Western blot) level to select candidates contributing to resistance. HRas, p38, CCL2, DOK1, PTK2B and JNK3 were integrated into a model of resistance-associated signalling alterations describing differential gene and protein expression between cisplatin-sensitive and -resistant cells in reaction to cisplatin exposure.

## 1. Introduction

Cisplatin is the backbone of treatment of non-small cell lung cancer (NSCLC). Patients with advanced or metastatic disease receive a cisplatin-based combination therapy if they carry neither an epithelial growth factor receptor (EGFR) nor an anaplastic lymphoma kinase (ALK) mutation [1]. One of the major drawbacks of this therapy is acquired resistance and the underlying mechanisms are multifactorial. In recent years, research has focused on cell signalling, as several pathways seem to play a major role in the development of chemoresistance. Signalling pathways, like extracellular-signal regulated kinase (ERK1/2), phosphatidylinositide-3-kinase/protein kinase B (PI3K/Akt) or P38 mitogen-activated protein kinases (p38MAPK) pathway are involved in the cellular reaction to cisplatin treatment [2,3]. Furthermore, tumour protein p53 (p53) signalling is a key pathway in apoptosis triggered by cisplatin [4] and p53 mutations are often associated with cisplatin resistance [5]. In a previous study, we found that cisplatin-resistant NSCLC cells are less susceptible to the drug-induced G_2_/M cell cycle arrest and apoptosis as compared to the sensitive counterparts [6].

Although many proteins and pathways relevant for cisplatin resistance have already been identified, a broader insight into its multi-mechanistic nature requires a system-wide screening approach. In the last decade, many high-throughput studies including functional screenings and genotyping investigations using genomics, proteomics and other “omics” technologies have been carried out [7]. Especially the latter genome-wide-scale experimental approaches provided a broader insight into the molecular mechanisms of cisplatin resistance. Among others, Galuzzi et al. [8] characterized the transcriptional response of A549 lung carcinoma cells to cisplatin in comparison with C2-ceramide and cadmium chloride, both inducers of mitochondrial apoptosis. Here, cisplatin showed a significantly different transcriptomic signature compared to the other two compounds. Toshimitsu et al. used the cDNA microarray technology to find 44 differentially expressed genes in sensitive and cisplatin-resistant oesophageal squamous cell carcinoma, among them 15 genes encoding ribosome-related proteins [9]. Gatti et al. identified several regulated pathways in response to cisplatin treatment in sensitive and resistant fission yeast strains [10]. The reaction to drug exposure was strain-specific: proteasome-mediated protein degradation, heat shock response and DNA repair were activated in cisplatin-sensitive yeast, whereas DNA mismatch repair, DNA damage recognition and cell cycle progression were the predominantly induced pathways in resistant counterparts [10]. Another study of Cheng et al. analysed multiple sensitive/resistant ovarian carcinoma cell line pairs using expression profiling [11]. The identified genes mostly belonged to cell surface interaction and trafficking pathways, which were not previously associated with cisplatin resistance. Here, none of the genes was differentially expressed in all six pairs of cells [11]. In a similar analysis by Yang et al. in seven different cancer cell line pairs representing four types of cancer, three pathways of differentially expressed genes were common for all pairs: leukocyte transendothelial migration, phosphatidylinositol signalling and cell adhesion molecules (CAMs) [12]. The authors compiled the 403 genes appearing in these pathways into a signalling network [12]. In another study of transcriptome alterations in cisplatin-resistant A549/CDDP cells compared to the sensitive ones, Yang et al. built up a systematic Ribonucleic acid (RNA)-network based on long noncoding RNA (lncRNA), protein-coding messenger RNA (mRNA) and microRNA (miRNA) [13]. A similar comparison was done by Hu et al., who analysed lncRNA and mRNA in A549 and the corresponding cisplatin-resistant variant A549/DDP. They revealed 67 differentially expressed pathways including p53 signalling and cell cycle regulation and generated a lncRNA-mRNA co-expression network [14]. Fang et al. also compared the A549 lung carcinoma cell line with a cisplatin-resistant counterpart A549/DDP using a transcriptome sequencing technique [15]. They identified in total 1214 differentially expressed genes. Most of these genes were enriched in the PI3K/AKT, mitogen-activated protein kinase, actin cytoskeleton regulation and focal adhesion pathways in the cisplatin-resistant subline [15].

Other groups focused on proteome profiling. Stewart et al. [16] identified 121 proteins differentially expressed in sensitive and cisplatin-resistant ovarian carcinoma cells and correlated protein expression with the respective mRNA levels. Another study utilized two-dimensional gel electrophoresis combined with mass spectrometry to identify 12 differentially expressed proteins in the comparison of cisplatin-sensitive and cisplatin-resistant A549/DDP lung carcinoma cells [17]. A recent report has presented a novel approach to correlate protein levels with cisplatin sensitivity based on microwestern arrays [18]. Such studies allow characterizing the effect of the drug on the cellular network of interactions in relation to cisplatin resistance.

Systems pharmacology is an emerging field considering not a single interaction between a drug and its target or pathway but the entire reaction of a cell to a drug exposure. A review by Wist et al. defined systems pharmacology as an approach, which develops a global understanding of the pathophysiology and the drug action on different organizational levels of a body system [19]. As the overall body system could be too complex to analyse it at once, the authors subdivided the body into an organ level, a tissue-cell level, an intracellular network level and a molecular level system, zooming from global more and more into detail. According to that understanding, we chose the intracellular network level as the starting point for our analysis.

As a cellular model, we used the human adenocarcinoma alveolar basal epithelial, non-small cell lung cancer cell line A549 and its cisplatin-resistant variant A549^r^CDDP^2000^ to identify key candidates involved in cellular response to cisplatin treatment. For this purpose, we applied a data-driven method using a whole genome array to investigate the entire transcriptome of our cell system after cisplatin exposure. After identifying key candidates based on differential expression and gene annotation, validation was performed via quantitative real-time polymerase chain reaction (qRT-PCR) and Western blot analysis.

## 2. Results

### 2.1. Workflow

As a starting point for a data-driven approach, we used a whole genome array. The cells were treated either with cisplatin or a drug-free medium (control cells). Cisplatin concentrations used were cell line-dependent and based on the respective EC_10_ (respective concentration leading to 10% of the maximum cytotoxic effect). This concentration enables assessing the transcriptional changes resulting from pre-apoptotic signalling rather than the degradative effects in nearly dead cells. Thus, the parental cells were treated with 11 µM cisplatin (EC_10_ of sensitive cell line). The resistant sub-line was exposed to 11 µM and additionally treated with 34 µM cisplatin (the respective EC_10_). In the following, “equimolar treatment” refers to the treatment of sensitive and resistant cell line with 11 µM cisplatin and ‘equitoxic treatment’ refers to the treatment of the sensitive cell line with 11 µM cisplatin and the resistant cell line with 34 µM cisplatin [6].

**Figure 1:**
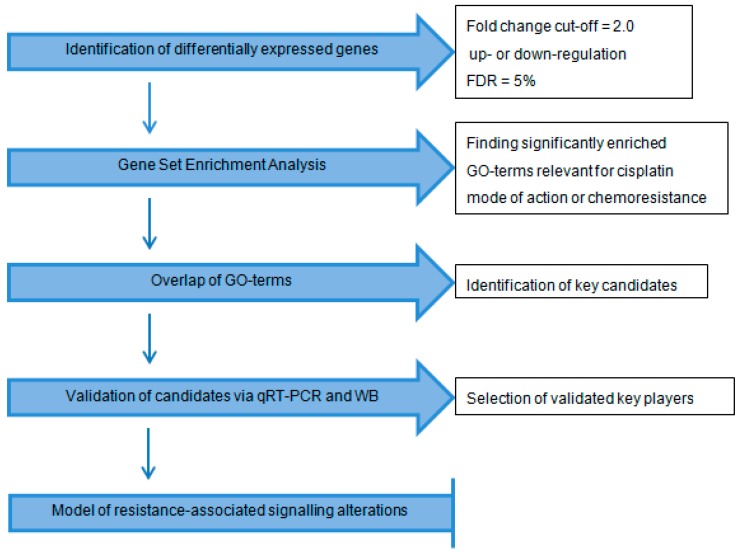
Flow chart of the array data processing (FDR: false discovery rate; GO: Gene Ontology; WB: Western blot).

The data were processed as shown in Figure 1: After extracting differentially expressed genes, a Gene Set Enrichment Analysis (GSEA) [20] was performed in order to identify key pathways altered in response to cisplatin treatment. The set of differentially expressed genes was then reduced to those involved in the identified pathways. The key candidates were validated using qRT-PCR and Western blot.

### 2.2. Microarray Analysis

#### 2.2.1. Differentially Expressed Genes

**Figure 2:**
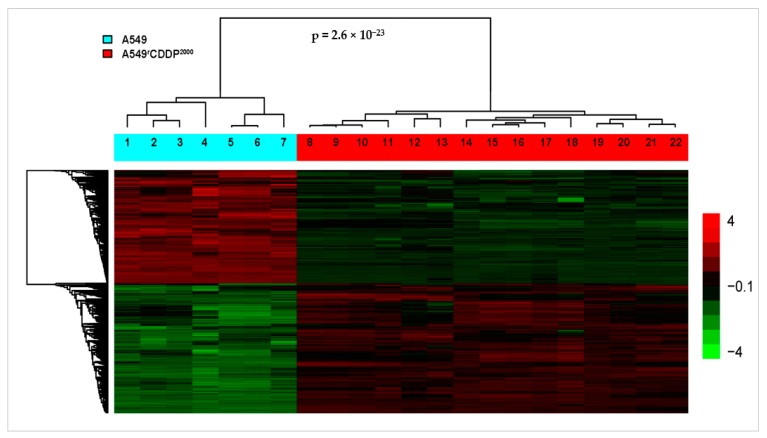
Heat map of the whole transcriptome, regulated genes with fold change cut-off at 2.0 and a false discovery rate of 5% of all replicates in sensitive and resistant cells. Numbers above lanes indicate: 1, 2, 3, 4: A549, untreated; 5, 6, 7: A549, treated with 11 µM cisplatin; 8, 9, 14, 15, 16: A549^r^CDDP^2000^, untreated; 10, 11, 17, 18, 19: A549^r^CDDP^2000^, treated with 11 µM cisplatin; 12, 13, 20, 21, 22: A549^r^CDDP^2000^, treated with 34 µM cisplatin. The *p*-value (2.6 × 10^−23^) corresponds to the result of a global test [21], which assesses the statistical significance of the entire signature that discriminates A549 and A549^r^CDDP^2000^ cells.

By using a whole genome array, we investigated the transcriptome of both cell lines in different treatment situations. The number of differentially expressed genes in the indicated comparisons of treatment situations with at least twofold up- or down-regulation and a false discovery rate of 5% in A549 and A549^r^CDDP^2000^ cells is presented in Table 1. The generated heat map of differentially expressed genes shows a clear clustering between the different treatment conditions and cell types based on an average linkage clustering using Pearson’s correlation distance (Figure 2).

The tree structure on top of the heat map indicates that A549 and A549^r^CDDP^2000^ cells cluster in two clearly separated groups. Interestingly, this separation is *not* due to cisplatin treatment but marks the differences between the cisplatin-sensitive vs. the cisplatin-resistant cell line. This shows that the adaptation to cisplatin over a long time changes the expression pattern much more than a single treatment with a higher dose. In the resistant cells, the difference in expression is dose-dependent, as cells treated with the higher dose cluster together. Furthermore, the number of differentially expressed genes caused by cisplatin treatment is larger in sensitive cells than in the resistant cells, even after exposure to the higher dose (Table 1). The technical validation of the microarray was performed by qRT-PCR with ten up- or down regulated genes in all different treatment conditions. The results of the qRT-PCR were consistent with the microarray data so that they were accepted as successfully validated.

#### 2.2.2. Gene Set Enrichment Analysis

After the identification of differentially expressed genes, a Gene Set Enrichment Analysis (GSEA) [20] was performed with respect to Gene Ontology (GO) terms [22] using HTSanalyzeR [23]. GSEA is a widely used method comparing the mapping of genes to a defined GO term with a ranking of these genes, e.g., via logarithmic fold change. The GSEA method calculates a score assessing the statistical significance of term enrichments with respect to the ranking of genes. More specifically, GSEA tries to reject the null hypothesis that genes belonging to a certain set of interest (e.g., specific GO biological process) are spread more or less uniformly all over the ranked list. On the other hand, a statistically enriched gene set corresponds to a comparably high fraction (larger than expected by chance) of its members appearing at the top or bottom of the ranked list.

Twelve GO terms were found to be statistically significant (FDR < 5%) associated with cisplatin treatment: actin filament bundle assembly, cell surface receptor signalling pathway, cytokine-mediated signalling pathway, cytoplasmic microtubule organization, hematopoietic progenitor cell differentiation, negative regulation of osteoblast differentiation, NOTCH receptor signalling, oocyte maturation, Ras protein signal transduction pathway, regulation of proteolysis, response to testosterone stimulus, vascular endothelial growth factor receptor (VEGFR) signalling pathway. The number of differentially expressed genes annotated with these twelve terms was far too large for further analysis. Therefore, we focused on those terms, for which a contribution to the mode of action of cisplatin or possible involvement in chemoresistance has been described in the literature, namely NOTCH receptor signalling [24,25], the VEGFR signalling pathway [26,27], the cell surface receptor signalling pathway [28,29] and the Ras protein signal transduction pathway [30,31].

**Figure 3:**
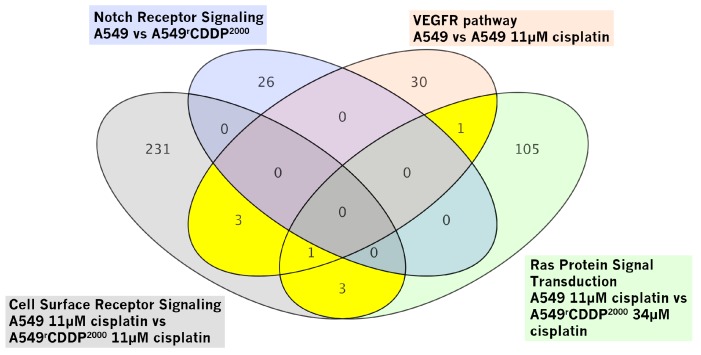
Venn diagram showing differentially expressed genes annotated with respective GO terms: The yellow sections indicate those genes, which were chosen for validation.

Interestingly, these four pathways were significantly enriched in different comparisons as indicated in Figure 3, e.g., the VEGFR pathway in treated with 11 µM cisplatin vs. untreated A549 cells. Importantly, the identified gene sets are not independent but share a number of differentially expressed genes. Numbers in the fields on the diagram indicate the number of genes, which were found in the indicated pathway. The yellow sections indicate those overlapping genes, which were considered for further analysis (Figure 3).

These shared genes comprise: *HRas, MAPK14* (p38α, further referred to as p38), C-C motif chemokine ligand 2 (*CCL2*), Docking protein 1 (*DOK1*), Docking protein 2 (*DOK2*), Protein tyrosine kinase 2 beta (*PTK2B*), *PTK2B* (highly similar transcript variant) and MAP kinase-activated protein kinase 2 (*MAPKAPK2*). For further investigation on mRNA and protein level, we decided to investigate only one isoform of DOK, DOK1, because of the high similarity between them. As MAPKAPK2 is directly associated downstream to p38 and directly regulated by p38 [32,33], we decided to analyse only p38 as the superordinate mitogen-activated protein kinase. Only one isoform of PTK2B was included in the validation. This data-driven method thus identified the following five key players for further evaluation: HRas, p38, CCL2, DOK1 and PTK2B.

### 2.3. Evaluation of the Identified Candidates

**Figure 4:**
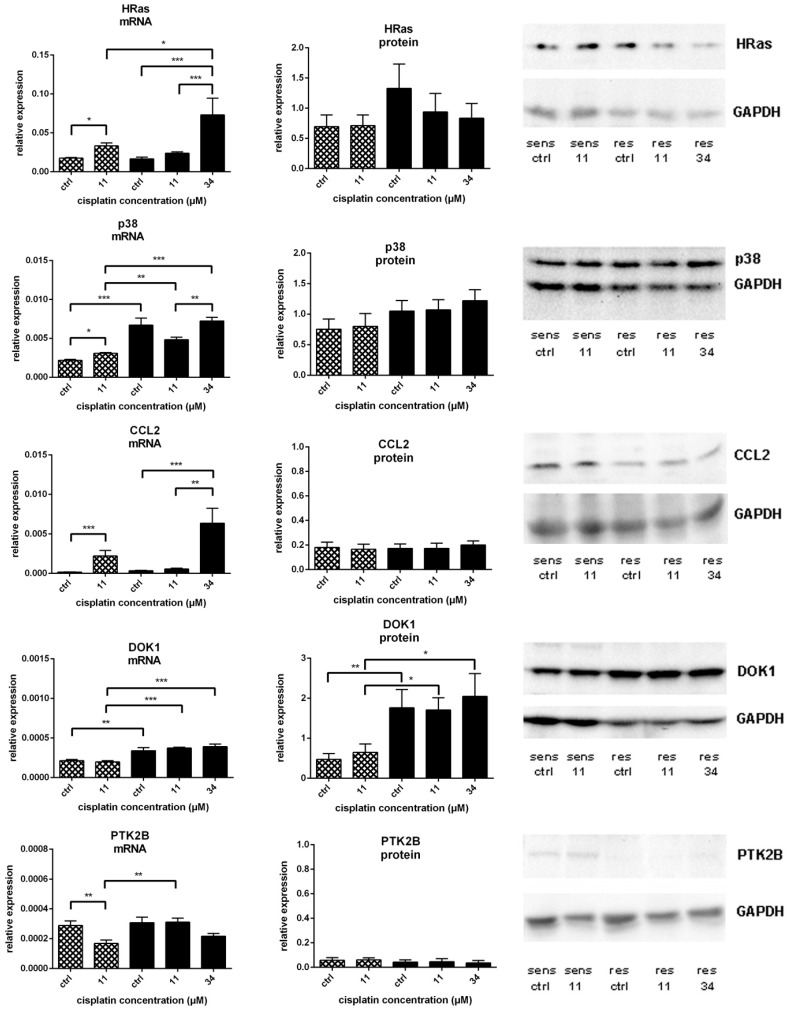
mRNA expression (all *n* = 6) of *HRas*, *MAPK14* (p38), *CCL2*, *DOK1* and *PTK2B* related to *GAPDH* mRNA expression; protein expression of HRas (*n* = 6), p38 (*n* = 6), CCL2 (*n* = 4), DOK1 (*n* = 7–8) and PTK2B (*n* = 3) related to GAPDH expression in A549 (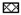) and A549^r^CDDP^2000^ (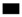) before (ctrl) and after treatment with 11 µM cisplatin (11) or 34 µM cisplatin (34) presented as mean ± SEM; as well as representative Western blots. * *p* < 0.05; ** *p* < 0.01; *** *p* < 0.01.

After the transcriptomic analysis, the validation of the identified genes was performed on the mRNA level by qRT-PCR and on protein level by Western blot analysis individually (Figure 4).

mRNA expression of *HRas*, a member of the oncogenic Ras family, was induced in both cell lines after exposure to the equitoxic concentrations of cisplatin but not in resistant cells after treatment with the equimolar concentration (11 µM). No significant changes were observed on protein level, although a slight decrease in HRas expression after exposure to 11 µM and 34 µM cisplatin was detected in A549^r^CDDP^2000^ cells (Figure 4).

*MAPK14* (p38), a kinase involved in stress response and cell cycle alterations, was also induced on mRNA level following cisplatin treatment but only in sensitive cells. Remarkably, the basal level of *MAPK14* (p38) mRNA was significantly higher in resistant cells than in the sensitive cell line. In A549^r^CDDP^2000^ cells, *MAPK14* (p38) expression was significantly elevated after equitoxic treatment (34 µM) compared to the equimolar concentration (11 µM cisplatin). These changes did not transfer to the protein level as only a slight and not significant increase in basal p38 expression was found in resistant cells compared to the sensitive ones (Figure 4).

*CCL2*, also known as *MCP-1* (monocyte chemotactic protein 1), a cytokine gene associated with invasion and metastasis, is connected to p38 [34,35]. Exposure to 11 µM cisplatin in A549 and to 34 µM cisplatin in A549^r^CDDP^2000^ cells significantly induced *CCL2* mRNA expression. Also in the case of *CCL2*, mRNA expression in resistant cells was significantly higher after equitoxic compared to equimolar treatment. No significant regulation could be observed on protein level after cisplatin exposure (Figure 4).

DOK1 is known as a tumour suppressor protein and a negative regulator of tyrosine kinases in mitogen-activated kinase signalling [36]. This candidate was not significantly influenced by cisplatin exposure. However, both mRNA and protein levels were significantly higher in the resistant cell line than in the sensitive counterpart, in untreated cells, as well as after equimolar and equitoxic treatment (Figure 4).

PTK2B, also referred to as Pyk2, promotes tumour proliferation through activation of MAPK signalling [37]. It also regulates response to cisplatin-induced stress through its interaction with p53 [38]. After cisplatin exposure, its expression was decreased on mRNA level in sensitive but not in resistant cells. *PTK2B* levels were therefore higher in the resistant cell line after equimolar treatment. A slight but not significant reduction in *PTK2B* mRNA was observed in A549^r^CDDP^2000^ cells after exposure to 34 µM cisplatin. No significant changes on protein level were found (Figure 4).

### 2.4. Extended Model of Resistance-Associated Signalling Alterations

**Figure 5:**
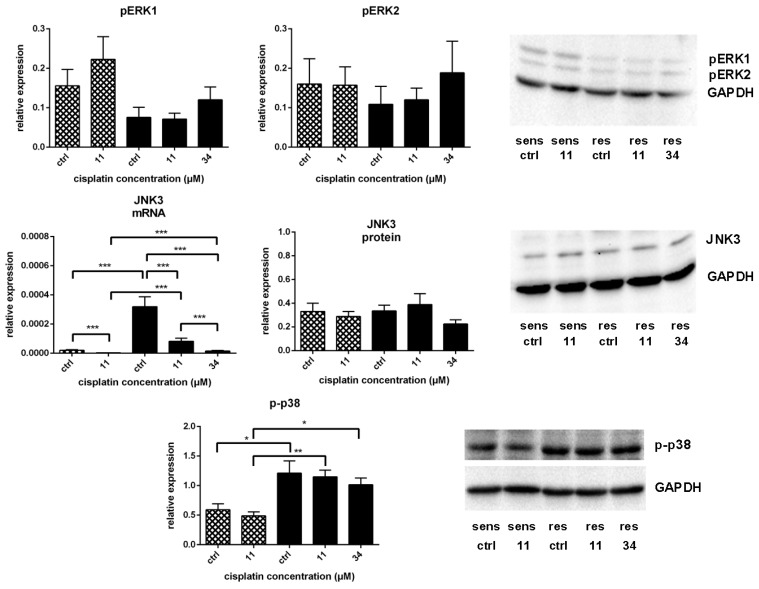
Expression of activated ERK (pERK1 and pERK2, both *n* = 7) related to GAPDH expression; mRNA expression of *JNK3* (*n* = 6) related to *GAPDH* mRNA expression; protein expression of JNK3 (*n* = 9) related to GAPDH expression; expression of activated p38 (p-p38, *n* = 4) related to GAPDH expression in A549 (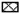) and A549^r^CDDP^2000^ (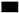) after treatment with 11 µM cisplatin (11) or 34 µM cisplatin (34) expressed as mean ± SEM, as well as representative Western blots.* *p* < 0.05; ** *p* < 0.01; *** *p* < 0.01.

After the evaluation of the key candidates, we took a closer look at the possible relationships between them based on the literature evidence. DOK1 is known as a negative regulator of Ras [36,39]. *HRas* belongs to the Ras oncogene family being central to the MAPK/ERK pathway [30]. However, the analysis of ERK activation revealed no significant differences between sensitive and resistant cell line (Figure 5).

On the other hand, HRas signalling reaches the nucleus via phosphorylation of c-Jun N-terminal kinases (JNKs) [40,41], which in turn is able to stabilize p53 by hindering mouse double minute 2 homolog (MDM2) binding, increasing p53 activation and supporting p53-induced apoptosis [42]. Additionally, JNKs appear to phosphorylate p53 at various sites after DNA damage [43].

We have recently documented a role of p53 in cisplatin resistance in A549^r^CDDP^2000^ cells and developed a model describing resistance-associated signalling alterations between the sensitive and resistant cell line [6]. It should be noted that JNKs can activate the p53 effector GADD45a (a growth arrest and DNA-damage inducible gene) also in the p53-independent manner [44]. For these reasons, it appeared interesting to assess the relevance of *JNK3*, which was found to be differentially regulated on the microarray, for cisplatin resistance in our cell model. Cisplatin treatment significantly reduced *JNK3* mRNA levels in sensitive and resistant cells (Figure 5). Interestingly, A549^r^CDDP^2000^ cells expressed significantly higher basal levels of *JNK3* mRNA compared to sensitive cells. Also after equimolar and equitoxic treatment, *JNK3* mRNA expression was elevated in the resistant cell line compared to the sensitive one. No significant regulation of JNK3 on protein level was observed, although a slight reduction in expression after treatment of resistant cells with 34 µM cisplatin could be detected (Figure 5). As no specific antibody for p-JNK3, which would not detect p-JNK1 and p-JNK2, is available, it was not possible to assess the activation status of this particular kinase.

PTK2B controls drug-induced apoptosis and cell survival by limiting p53 levels [38]. Another important but positive, regulator of p53 is p38. It plays a key role in the induction of p53-mediated apoptosis by chemotherapeutic agents including cisplatin [45]. Therefore, we examined phosphorylation of p38 in both cell lines before and after cisplatin treatment. As mentioned above, the total protein expression of p38 was not different in sensitive and resistant cells and was not influenced by cisplatin. As is clear from Figure 5, the platinum drug did not activate p38 in either cell line. Nevertheless, basal phosphorylation level was significantly higher in A549^r^CDDP^2000^ cells. It was also the case after equimolar and equitoxic treatment (Figure 5).

The interaction between p38 and CCL2 is bidirectional. On one hand, CCL2 was reported to activate p38 [34]. In our case, cisplatin induced *CCL2* expression in sensitive cells, which however did not result in p38 activation. On the other hand, there is evidence that *CCL2* expression is controlled by p38 [35]. This is well in agreement with our data, as cisplatin exposure enhanced both p38 (*MAPK14*) and *CCL2* mRNA expression in sensitive cells.

**Figure 6:**
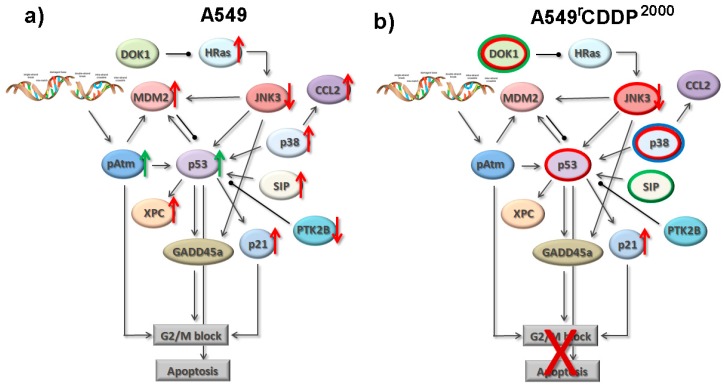
Model of resistance-associated signalling alterations indicating significant changes of mRNA expression (red arrows) or protein expression (green arrows) after cisplatin treatment in A549 and A549^r^CDDP^2000^ cells, as well as an increase in basal mRNA levels (red circles), protein levels (green circles) or basal kinase activation (blue circles) in A549^r^CDDP^2000^ (**b**) compared to A549 (**a**) cells. The model is based on the data presented and described here and the data previously published [6].

Based on the findings and literature evidence presented above we have extended the previous model [6] summarizing the interactions between the identified candidates (Figure 6). In addition to our previous work [6], we have now included the significant differences in basal mRNA or protein expression, or kinase activation (indicated as red, green or blue circles, respectively) between sensitive and resistant cells into the model as these are also relevant for cisplatin resistance as discussed below. The model is based on the different reaction of A549 and A549^r^CDDP^2000^ cells to the equimolar concentrations of cisplatin.

## 3. Discussion

### 3.1. Systems Pharmacology Approach

Earlier studies have clearly pointed out that a systems approach to address the problem of chemoresistance to cisplatin has advantages over the conventional target-centred methodology, which mainly identifies single proteins or a list of affected pathways [2,3,46] without displaying any functional connections. Our previous work [6] utilized a common hypothesis-driven approach to characterize cisplatin resistance in NSCLC cells and establish connections based on the literature evidence. Here, we have followed a data-driven top-down approach, which involves iterative filtering of the massive amount of data of the whole genome microarray. This was done by statistical means without limiting the results by a predefined hypothesis. Here, the reduction was done by choosing those differentially expressed genes, which occurred simultaneously in different GO terms. This increased the a priori chance of these genes to play a major role in cisplatin resistance. On the other hand, this way of analysis could have led to the loss of relevant genes.

Nevertheless, by analysing not only the mRNA levels in a broad whole transcriptome approach but adding the protein levels of the identified key candidates we could extend our previously developed model of resistance-associated alterations [6]. As mentioned above, some high-content analyses of cisplatin resistance using different techniques were performed in the last years. Galuzzi et al. studied transcriptional alterations following cisplatin exposure in drug-sensitive A549 NSCLC cells without comparing it with the corresponding resistant variant and analysing the connections between the identified genes [8]. The identified cisplatin-induced modulations are different from our findings. Noteworthy, the authors reported a surprisingly little overlap between their results [8] and CDDP response modifiers found in a genome-wide siRNA screening in the same cells [47].

In an attempt to identify resistance-specific signatures, Yang et al. analysed the RNA expression profiles of A549 and cisplatin-resistant A549/DDP lung carcinoma cells. They observed a clear hierarchical clustering of the two cell lines, which is in agreement with our data. The validation by qRT-PCR was performed on eight mRNAs implicated to be important for cisplatin resistance [13]. Interestingly, some of the significantly enriched signalling pathways from Yang et al. like MAPK signalling [13] are similar to those identified in our study. Besides identifying a great number of mRNAs differentially expressed in A549 and A549/CDDP cells, the authors conclude that cisplatin resistance is also related to changes in long non-coding (lnc) RNAs. They also built up a signalling network of those specific RNAs [13]. The importance of lncRNA was also highlighted by Hu et al. who showed that knockdown of the aberrantly expressed lncRNA UCA1 sensitized resistant A549/DDP cells to cisplatin [14]. The identified resistance-specific pathways like p53 signalling and cell cycle correlate well with the results of our previous work [6].

Interestingly, in a comprehensive evaluation of seven sensitive/resistant cell line pairs a clear clustering was observed for each pair [12]. The differentially regulated pathways common in all pairs were different from those found in our study [12]. Nevertheless, the network included several genes found relevant in our case, among them *MDM2, PTK2B* and p38 *MAPK*s. In contrast, Fang et al. [15] identified MAPK signalling as one of the pathways significantly altered in cisplatin-resistant A549/DDP cells, which agrees well with our results and the findings of Yang et al. [13].

However, the effect of cisplatin on the RNA expression was not investigated in most studies. Only Gatti et al. examined the effect of the platinum drug on the sensitive and resistant fission yeast strains and documented strain-specific drug response [10]. Our findings show that not only the differences in the basal gene or protein expression account for resistance but also the drug effect is altered in resistant cells. Studying how a drug influences a whole cell system is a focus of systems pharmacology. In that respect, we have done the first step towards the systems pharmacology approach in our cell model.

Zeng et al. compared the proteome of A549 cells and cisplatin-resistant A549/CDDP cells using a combination of two-dimensional gel electrophoresis and mass spectrometry and identified twelve resistance-related proteins, without compiling the data in a network and discussing interactions [17]. The identified proteins were different from our results, as the authors analysed the proteome level and we based our protein analysis on previously found differences on mRNA level. It should also be noted that the identification of differentially expressed proteins in the gel is intrinsically limited to the abundant proteins as those are the most likely to be detected.

Furthermore, most studies did not include a robust validation of the identified targets on the protein level. As is clear from our data, and has already been suggested by Stewart et al., changes in mRNA expression do not necessarily correlate with protein abundance highlighting the need for validation studies on both mRNA and protein level [16]. The authors carried out proteome profiling of sensitive and cisplatin-resistant ovarian carcinoma cells and correlated the results with mRNA expression profiles. They found a discrepancy between mRNA and protein expression in more than the half of the proteome indicating an important role of posttranscriptional regulation in controlling protein expression [16]. Our findings also show a weak correlation between the mRNA and protein data. This can be attributed to the choice of the time point of the measurement, to posttranscriptional and posttranslational factors. The influence of translational efficiency and protein half-life on the correlation between mRNA and protein levels is likely to be different for each gene and respective protein demanding the systematic assessment of expression regulation [48]. Proteome profiling using microwestern arrays [18] opens new opportunities to perform large-scale correlation studies between gene and protein expression in context of cisplatin resistance. The recent report by Stark et al. [18] also highlighted the importance of duration of cisplatin exposure for protein levels as the differences in protein expression between sensitive and resistant cells altered greatly over time.

To the best of our knowledge, we are the first to investigate the reaction of resistant cells to equimolar and equitoxic concentrations of cisplatin. The differences in the effect of the equimolar concentrations on A549 and A549^r^CDDP^2000^ cells reflect the mechanisms of resistance. In most cases, sensitive and resistant cells reacted similarly to the equitoxic concentrations. The absence of p38 and PTK2B regulation in A549^r^CDDP^2000^ cells after treatment with 34 µM cisplatin is intriguing given the same growth inhibitory effect in both cell lines. This underscores the fundamental differences between the sensitive and resistant phenotype, which was also evident from the clear clustering of the two cell lines on the heat map of differentially expressed genes.

### 3.2. Role of the Identified Key Players

Cisplatin leads to DNA damage through the formation of DNA adducts. This toxic insult triggers activation of several different pathways for survival or apoptosis, depending on the amount of DNA damage. In chemoresistant cancer cells, these pathways appear to be significantly dysregulated.

This work identified HRas, p38, CCL2, DOK1, PTK2B and JNK3 as key players of cisplatin resistance. *HRas* is one of the genes of the Ras oncogenic family. Activating *Ras* mutations in several cancer entities were held responsible for tumour development [49,50]. The connection between HRas and cisplatin resistance was established already many years ago [51]. The absence of *HRas* regulation after equimolar treatment in resistant cells may be a consequence of the elevated DOK1 expression as DOK1 is a negative regulator of Ras [36,39]. One could expect that cisplatin-induced increase in *HRas* expression in sensitive cells would result in higher JNK3 levels, which in turn would lead to an activation of p53. However, cisplatin triggered down-regulation of JNK3 in both cell lines. Whereas some studies documented the role of JNK signalling in p53 activation [42,43], others suggested the JNK pathway to be a negative regulator of p53 [52]. Interestingly, A549^r^CDDP^2000^ cells feature higher basal mRNA levels of *JNK3* and *TP53* (p53). It was shown in mantle cell lymphoma that consecutive expression of JNK is required to promote proliferation [53]. Overexpression of *TP53* (p53) mRNA is common in cisplatin-resistant cancer cell lines [54] and was reported to correlate with resistance to the drug in lung carcinoma patient samples [55]. It has been suggested that *TP53* (p53) in resistant cells is often mutated resulting in the loss of function, thus, not being able to mediate apoptosis [5].

PTK2B controls p53 through the regulation of the MDM2-associated p53 turnover. Its knockdown was reported to increase p53 levels and inhibit cell proliferation [38], which is in agreement with our results. On the other hand, the expression of PTK2B active domain in human fibroblasts blocked cisplatin-induced apoptosis [38].

Another upstream regulator of p53 is p38. Cisplatin exposure increased *MAPK14* (p38) mRNA expression in sensitive cells but not in resistant counterparts. However, the drug failed to activate p38 in both cell lines. Noteworthy, we detected the significantly higher basal levels of *MAPK14* (p38) mRNA and an increase in basal p38 activation in A549^r^CDDP^2000^ cells, which is not uncommon. Fang et al. observed increased expression of genes belonging to p38 MAPK pathway in cisplatin-resistant A549/DDP cells compared to the parent cell line [15]. High levels of p38 have been associated with poor prognosis in other cancer entities [56]. Inhibition of p38 activation was reported to sensitize tumour cells to cisplatin and etoposide [57,58].

As p38 regulates CCL2 expression [35], the observed increased *CCL2* levels after cisplatin exposure in sensitive cells but not in resistant cells were expected. These results suggest the relevance of CCL2 for cisplatin resistance in our cell model. A previous study showed that expression of CCL2 in ovarian cancer cells correlates with chemotherapy response and is reduced in cisplatin-resistant cells [59]. Another study revealed that *CC*L2 expression is induced after treatment with cisplatin [60], which is in agreement with our data.

### 3.3. Model of Resistance-Associated Signalling Alterations

Based on our results presented above, we built a preliminary signalling network, which can explain the different reaction of the sensitive and resistant cell lines to cisplatin treatment (Figure 6). Within the model we display possible connections between the identified key players, which could serve as a basis for further hypotheses and investigations of the proteome. This could be limited by the fact that the model is far not comprehensive and needs to be extended by further proteins, which could additionally account for the effects on cell cycle and apoptosis. In our study, we had to reduce the number of candidate genes and did not explore distinct perturbations of the signalling network. On the other hand, the strength of the model is that the alterations in the gene and protein expression are all based on experimental data. Our future work will focus on the validation and extension of the model. Furthermore, the dynamic nature of mRNA and protein expression makes it necessary to integrate the temporal dimension into the model.

At the end, our model aims at depicting the whole proteome and transcriptome allowing the description of the response of all relevant signalling pathways to cisplatin exposure. Mathematical models could make it possible to forecast the outcome of specific perturbations of the network serving as a biomarker for chemotherapeutic response.

## 4. Materials and Methods 

### 4.1. Drugs

Cis-diamminedichloroplatinum (II) (cisplatin) was obtained from Sigma–Aldrich, Steinheim, Germany and dissolved in 0.9% sodium chloride (NaCl) to a concentration of 1.5 g/L. Aliquots were stored at −20 °C and thawed immediately before use. Each aliquot was used only once. Cisplatin cytotoxicity was assessed according to the previously described procedure [6] using freshly prepared cisplatin solutions (A549: pEC_50_ = 4.500 ± 0.042, A549^r^CDDP^2000^: pEC_50_ = 4.307 ± 0.030, mean ± SD, *n* = 4) and compared to our previously published data obtained with aliquots of cisplatin solutions (A549: pEC_50_ = 4.522 ± 0.144, *n* = 11, A549^r^CDDP^2000^: pEC_50_ = 4.262 ± 0.171, *n* = 12, mean ± SD [6]). Cytotoxicity was not significantly different (*p* > 0.05) and thus not influenced by the preparation procedure. Individual pEC_50_ values for each independent experiment with freshly prepared cisplatin solutions are summarized in Supplementary Table S1.

### 4.2. Cell Lines

The human NSCLC cell line A549 was obtained from American Type Culture Collection (ATCC) (Manassas, VA, USA). Its cisplatin-resistant sub-line A549^r^CDDP^2000^ derived from the Resistant Cancer Cell Line (RCCL) collection (www.kent.ac.uk/stms/cmp/RCCL/RCCLabout.html) had been established by adapting the growth of A549 cells in the presence of increasing concentrations of cisplatin until a final concentration of 2000 ng/mL cisplatin as described previously [61]. A549 cells were grown in IMDM medium (PAN-Biotech, Aidenbach, Germany) containing 4 mM L-glutamine supplemented with 10% foetal calf serum, 100 I.E./mL penicillin and 0.1 mg/mL streptomycin. The medium of the A549^r^CDDP^2000^ cells additionally contained 2 µg/mL cisplatin. Cells were cultivated as monolayers in a humidified atmosphere at 37 °C and 5% CO_2_. Every ten passages, a new backup of cells was thawed to ensure the reproducibility of the results. For all experiments, cells were allowed to attach overnight, experienced 4 h of serum starvation and were subsequently treated with cisplatin for 24 h in IMDM medium without any supplements. The parental cells were treated with 11 µM cisplatin. The resistant sub-line was exposed to 11 µM cisplatin and additionally treated with 34 µM cisplatin. The control cells were treated with the drug-free medium. Prior serum starvation and the use of the unsupplemented medium allowed us to avoid the influence of growth factors in serum on signalling response.

### 4.3. Microarray

Total ribonucleic acid (RNA) was isolated from the cells with my-Budget RNA Mini Kit (Bio-Budget, Krefeld, Germany) through different spin columns according to the manufacturer’s instructions. Isolated RNA was stored at −80 °C until analysis was performed.

Transcriptome was then analysed using One-Color Whole Genome Array SurePrint G3 Human GE V2 8x60K Kit (Agilent Technologies, Santa Clara, CA, USA) according to the manufacturer’s instructions. Briefly, total RNA was transcribed to complementary deoxyribonucleic acid (cDNA) using AffinityScript-RT, Oligo dT-Promoter Primer and T7 RNA Polymerase and labelled using the One Colour RNA Spike-In Kit (positive controls) including Cyanin 3-CTP (Cy3) dye. After purifying the labelled/amplified complementary RNA (cRNA) using silica-membrane RNeasy spin columns from the RNeasy^®^ Mini Kit (Qiagen, Venlo, the Netherlands), cRNA was quantified spectrophotometrically using NanoDrop^TM^ ND-1000 (Thermo Fisher Scientific Inc., Waltham, MA, USA). 40 µL of equivalent amounts of Cy3-labelled cRNA in 10× Blocking Agent and 25× Fragmentation Buffer, diluted with 2× GEx HI-RPM Hybridization Buffer were loaded on the gaskets of the microarray slide and kept at 65 °C for 17 h with 10 rpm of agitation. After washing twice with different washing buffers, the microarray was read out with the SureScan Microarray Scanner System (Agilent Technologies, Santa Clara, CA, USA) to obtain immunofluorescence intensity. Array data were pre-processed via background correction (exponential convolution method) [62] and quantile normalization [63]. Statistical significances of dose- and resistance-induced gene expression changes were analysed using limma (Linear Models for Microarray Data, Bioconductor version 3.6, open source software for bioinformatics) [64], a linear model-based technique. Differential expression was declared at a 5% false discovery rate (FDR) cut-off together with an at least twofold up- or downregulation. The overall significance of the signature of differentially expressed genes was assessed via a global test [21]. The microarray data have been deposited into the Gene Expression Omnibus database under the accession number GSE108214.

### 4.4. RNA Isolation, cDNA Synthesis and qRT-PCR

Whole cell RNA was isolated after treatment using the my-Budget RNAse Mini Kit (Bio-Budget, Krefeld, Germany) and quantified spectrophotometrically with a NanoDrop^TM^ N-1000 (Thermo Fisher Scientific Inc., Waltham, MA, USA). Subsequent cDNA synthesis was performed for 60 min at 42 °C. The reaction mixture was composed of 2 µL water, 1.5 µL 10× buffer (Life Technologies, Carlsbad, CA, USA), 1.1 µL MgCl_2_ solution (25 mM; Life Technologies, Carlsbad, CA, USA), 1.5 µL dithiothreitol solution (100 mM; Life Technologies, Carlsbad, CA, USA), 1.5 µL dNTP (2.5 mM; Life Technologies, Carlsbad, CA, USA), 0.6 µL Rnasin^®^ (20 U/µL; Life Technologies, Carlsbad, CA, USA), 0.3 µL oligo-dT-primer (Life Technologies, Carlsbad, CA, USA) and murine leukaemia virus reverse transcriptase (50 U/µL Life Technologies, Carlsbad, CA, USA). qRT-PCR was performed according to the manufacturer’s instructions using the LightCycler^®^ 480 SYBR Green I Master (Hoffmann La Roche, Basel, Switzerland). Primers (GTPase HRas: forward 5′-TGGACGAATACGACCCCACT-3′, reverse 5′-CCAACGTGTAGAAGGCATCC-3′; Mitogen-activated protein kinase 10 (JNK3): forward 5′-AAGCACCTCCATTCTGCTGG-3′, reverse 5′-GGAAGGTGAGTCCCGCATAC-3′; P38 mitogen-activated protein kinases (p38): forward 5′-TGCCGCTGGAAAATGTCTCA-3′, reverse 5′-GTTGTTCAGATCTGCCCCCA-3′; C-C motif chemokine ligand 2 (CCL2): forward 5′-CGCCTCCAGCATGAAAGTCT-3′, reverse 5′-TGTCTGGGGAAAGCTAGGGG-3′; protein tyrosine kinase 2 Beta (PTK2B): forward 5′-AAGGACATTGCCATGGAGCA-3′, reverse 5′-TGACCTTTTCAGCCTCCCAC-3′; Docking protein 1 (DOK1): forward 5′-TCTACCTGAGAAGGACGGCA-3′, reverse 5′-TCCAGGCACAGTCCAACATC-3′, annealing temperature 60 °C) were purchased from Life Technologies, Carlsbad, CA, USA. Quality of the qRT-PCR was proved by recording the melting curve of the DNA products. PCR experiments were repeated six times for each gene. The results of individual experiments are presented in Supplementary Table S2.

### 4.5. SDS-PAGE and Western Blot

Cellular proteins were extracted using RIPA buffer (50 mM Tris-HCl (pH 7.6), 150 mM NaCl, 1% Triton X-100, 1% sodium desoxycholate, 0.1% SDS, 1 mM EDTA) with protease inhibitors (2 µM pepstatin, 2 µM leupeptin, protease inhibitor cocktail (Sigma-Aldrich, Steinheim, Germany), 1 mM activated Na_3_VO_4_, 1 mM NaF). Protein concentrations were determined with the bicinchoninic acid assay (BCA, Merck KGaA, Darmstadt, Germany). Whole protein extracts were separated using polyacrylamide gel electrophoresis. Proteins were transferred to a PVDF membrane (Roti^®^-PVDF, Carl Roth, Karlsruhe, Germany), which was blocked after protein transfer with 5% (*w*/*v*) not-fat dry milk powder in Tris-buffered saline (TBS) with 0.1% (*v*/*v*) Tween-20 (TBS-T) for 1 h at room temperature. Subsequently, the membranes were incubated overnight at 4 °C with primary goat antibodies diluted in TBS-T (anti-HRas GTX-116041, 1:500; anti-JNK3 GTX-103148, 1:1000; anti-p38α GTX-110720, 1:500; anti-DOK1 GTX-101610, 1:500, all GeneTex, Irvine, CA, USA; anti-CCL2 AVARP07046, 1:2000, Aviva Systems Biology, San Diego, CA, USA; PTK2B H00002185-M01: 1:500, Abnova Corporation, Taipei City, Taiwan; anti-p-p38α BYT-ORB6578, 1:1000, Biorbyt, Cambridge, UK) and washed three times for 10 min with TBS-T followed by incubation with a primary goat antibody against glyceraldehyde 3-phosphate dehydrogenase (GAPDH) diluted in TBS-T (GTX100118, 1:20,000, GeneTex, Irvine, CA, USA) for 30 min. at room temperature. After the washing steps the membranes were incubated with a secondary HRP-conjugated antibody (goat anti-rabbit IgG-HRP SBA-4030-05, diluted 1:1000 in TBS-T, Southern Biotech, Birmingham, AL, USA) for 1 h at room temperature. The detection was performed with Enhanced Chemoluminescence (ECL) reagent (Pierce™ ECL Western Blotting Substrate, Thermo Fisher Scientific Inc., Waltham, MA, USA) on a Molecular Imager ChemiDoc^TM^ XRS+ System from Bio-Rad Laboratories GmbH, Munich, Germany. Densitometric analysis was carried out using Image Lab^TM^ Software 6 (6.0, Bio-Rad Laboratories, Hercules, CA, USA) based on the results of three to nine experiments as stated in the respective figure legends. The results of individual experiments are presented in Supplementary Tables S3 and S4.

### 4.6. Statistical Analysis

All statistical analyses were performed using Prism^®^ V6 (GraphPad Software, La Jolla, CA, USA). Significance of differences in expression of the identified candidates was analysed based on logarithmic values using a one-way analysis of variance (ANOVA) with a Holm-Sidak post-test. Differences were considered to be statistically significant at *p*-value < 0.05.

## 5. Conclusions

This study identified several key players, such as HRas, p38, CCL2, DOK1, PTK2B and JNK3, involved in the mechanisms of cisplatin resistance in NSCLC cells. In a model of signalling alterations, we describe interactions of various proteins associated with cisplatin resistance and provide a hypothesis how differences in their regulation may lead to the lack of cell cycle arrest and apoptosis in resistant cells. Thus, our study can be regarded as the first step towards a systems pharmacology approach, characterizing the reaction of the cellular system to cisplatin in the context of cisplatin resistance.

## Figures and Tables

**Table 1:** Number of differentially expressed genes, compared as treatment condition 1 vs. condition 2 with at least twofold up- or down-regulation and a false discovery rate of 5%.

Treatment Condition 1	Treatment Condition 2	Number of Differentially Expressed Genes
A549, untreated	A549^r^CDDP^2000^, untreated	3697
A549, 11 µM cisplatin	A549^r^CDDP^2000^, 11 µM cisplatin	4394
A549^r^CDDP^2000^, untreated	A549^r^CDDP^2000^, 11 µM cisplatin	27
A549^r^CDDP^2000^, untreated	A549^r^CDDP^2000^, 34 µM cisplatin	708
A549, untreated	A549, 11 µM cisplatin	1191
A549, 11 µM cisplatin	A549^r^CDDP^2000^, 34 µM cisplatin	3670

## Supplementary Materials

Supplementary materials can be found at http://www.mdpi.com/1422-0067/19/3/767/s1.

## Abbreviations

NSCLC	non-small cell lung cancer
CDDP, DDP	cis-diamminedichloroplatinum (II) (cisplatin)
GO	Gene Ontology
GSEA	Gene Set Enrichment Analysis
FDR	false discovery rate
VEGFR	vascular endothelial growth factor receptor
TBS	Tris-buffered saline
MAPK	mitogen-activated protein kinase
ERK	Extracellular-signal Regulated Kinase
DOK1	Docking protein 1
PTK2B, Pyk2	Protein tyrosine kinase 2 beta
CCL2	C-C motif chemokine ligand 2
MCP-1	monocyte chemotactic protein 1
MAPKAPK2	MAP kinase-activated protein kinase 2
JNK	c-Jun N-terminal kinase
MDM2	mouse double minute 2 homolog
GAPDH	glyceraldehyde 3-phosphate dehydrogenase
