# Supplementary material for: Key Players of Cisplatin Resistance: Towards a Systems Pharmacology Approach
Source: Int J Mol Sci. 2018 Mar 7;19(3):767. doi: 10.3390/ijms19030767 (PMC5877628; doi:10.3390/ijms19030767)
Supplement: Supplementary file 1 [file ijms-19-00767-s001.pdf]

Key players of cisplatin resistance: towards a systems pharmacology approach

Navin Sarin, Florian Engel, Florian Rothweiler, Jindrich Cinatl jr., Martin Michaelis, Roland Frötschl, Holger Fröhlich and Ganna V. Kalayda*

Supplementary Information

Supplementary Table S1. Cisplatin cytotoxicity in A549 and A549^{r}CDDP^{2000} cells assessed as described previously [6] using freshly prepared cisplatin solutions: pEC$_{50}$ values of individual experiments.

	**A549**	**A549^{r}CDDP^{2000}**
pEC$_{50}$	4.480	4.309
	4.558	4.348
	4.501	4.292
	4.460	4.278

Supplementary Table S2. mRNA expression of the genes under study related to GAPDH mRNA expression in A549 and A549rCDDP2000 cells before (ctrl) and after treatment with 11 µM cisplatin (11) or 34 µM cisplatin (34): results of individual experiments.

Gene	A549, ctrl	A549, 11	A549rCDDP2000, ctrl	A549rCDDP2000, 11	A549rCDDP2000, 34
HRas	0.0195	0.0433	0.0146	0.0213	0.0480
	0.0156	0.0461	0.0131	0.0237	0.0439
	0.0212	0.0278	0.0117	0.0310	0.1680
	0.0158	0.0272	0.0140	0.0256	0.1020
	0.0163	0.0268	0.0268	0.0218	0.0398
	0.0160	0.0282	0.0188	0.0187	0.0361
p38	0.00261	0.00352	0.00719	0.00580	0.00770
	0.00217	0.00300	0.00652	0.00564	0.00770
	0.00186	0.00292	0.00391	0.00396	0.00843
	0.00182	0.00269	0.00439	0.00399	0.00798
	0.00204	0.00317	0.00873	0.00501	0.00609
	0.00252	0.00313	0.00942	0.00461	0.00552
CCL2	0.0000805	0.0015324	0.0004556	0.0006624	0.0077585
	0.0000906	0.0016653	0.0005055	0.0007715	0.0075989
	0.0002672	0.0041866	0.0002458	0.0006310	0.0103804
	0.0002326	0.0044871	0.0003069	0.0007299	0.0109722
	0.0001212	0.0006488	0.0002425	0.0002294	0.0005807
	0.0001273	0.0006095	0.0002599	0.0002294	0.0006310
DOK1	0.000190	0.000181	0.000367	0.000388	0.000485
	0.000205	0.000185	0.000446	0.000397	0.000391
	0.000248	0.000170	0.000189	0.000380	0.000280
	0.000196	0.000156	0.000242	0.000373	0.000309
	0.000175	0.000248	0.000408	0.000378	0.000428
	0.000262	0.000242	0.000373	0.000313	0.000446
PTK2B	0.0002185	0.0002672	0.0002690	0.0004310	0.0002155
	0.0004163	0.0001393	0.0004021	0.0003501	0.0002709
	0.0003090	0.0001345	0.0001750	0.0002617	0.0002025
	0.0003090	0.0001179	0.0002563	0.0002804	0.0001402
	0.0002262	0.0001589	0.0003599	0.0002617	0.0002231
	0.0002563	0.0001929	0.0003858	0.0002804	0.0002510
JNK3	0.0000136	0.0000016	0.0003675	0.0000912	0.0000104
	0.0000080	0.0000022	0.0002985	0.0000912	0.0000112
	0.0000159	0.0000010	0.0001116	0.0000201	0.0000116
	0.0000187	0.0000049	0.0001482	0.0000281	0.0000049
	0.0000285	0.0000013	0.0004652	0.0001116	0.0000285
	0.0000343	0.0000012	0.0005233	0.0001492	0.0000208

Supplementary Table S3. Protein expression of the total proteins under study related to GAPDH expression in A549 and A549^{r}CDDP^{2000} cells before (ctrl) and after treatment with 11 μM cisplatin (11) or 34 μM cisplatin (34): results of individual experiments.

Gene	A549, ctrl	A549, 11	A549^{r}CDDP^{2000}, ctrl	A549^{r}CDDP^{2000}, 11	A549^{r}CDDP^{2000}, 34
HRas	0.13	0.16	0.15	0.14	0.11
	0.12	0.19	0.2	0.14	0.15
	0.77	0.92	1.21	0.86	0.8
	1.19	0.91	1.69	1.47	1.52
	0.8	1.21	2.24	0.93	1.15
	1.15	0.87	2.46	2.07	1.27
p38	0.39	0.36	0.47	0.57	0.58
	0.48	0.40	0.72	0.69	0.84
	0.62	0.59	1.35	1.57	1.75
	0.6	0.65	0.94	0.9	1.22
	1.48	1.69	1.63	1.3	1.51
	0.96	1.11	1.19	1.39	1.42
CCL2	0.11	0.11	0.1	0.09	0.15
	0.1	0.08	0.13	0.11	0.15
	0.27	0.26	0.26	0.25	0.29
	0.24	0.21	0.2	0.24	0.21
DOK1			1.13	1.55	2.33
	0.6	0.77	1.4	1.48	1.57
	1.2	0.84	2.66	3.09	4.74
	0.63	0.64	1.35	1.58	1.44
	0.07	0.14	1.2	1.68	0.97
	0.26	1.71	4.63	2.81	4.23
	0.31	0.17	0.99	1	0.41
	0.21	0.25	0.7	0.44	0.65
PTK2B	0.02	0.03	0.01	0.01	0.01
	0.10	0.09	0.07	0.09	0.08
	0.05	0.06	0.05	0.04	0.02
JNK3	0.54	0.36	0.37	0.28	0.27
	0.63	0.38	0.59	0.91	0.34
	0.2	0.13	0.21	0.18	0.1
	0.21	0.16	0.32	0.21	0.23
	0.64	0.51	0.53	0.78	0.38
	0.18	0.24	0.32	0.2	0.11
	0.28	0.36	0.29	0.42	0.21
	0.11	0.18	0.15	0.15	0.1
	0.18	0.27	0.23	0.37	0.28

Supplementary Table S4. Protein expression of the phosphorylated proteins under study related to GAPDH expression in A549 and A549^{r}CDDP^{2000} cells before (ctrl) and after treatment with 11 μM cisplatin (11) or 34 μM cisplatin (34): results of individual experiments.

Gene	A549, ctrl	A549, 11	A549^{r}CDDP^{2000}, ctrl	A549^{r}CDDP^{2000}, 11	A549^{r}CDDP^{2000}, 34
pERK1	0.05	0.07	0.01	0.01	0.03
	0.22	0.22	0.06	0.09	0.17
	0.06	0.51	0.03	0.04	0.2
	0.21	0.2	0.03	0.08	0.09
	0.02	0.07	0.06	0.06	0.07
	0.3	0.31	0.19	0.13	0.03
	0.23	0.18	0.15	0.09	0.25
pERK2	0.02	0.06	0.01	0.02	0.05
	0.1	0.13	0.1	0.21	0.21
	0.11	0.36	0.03	0.06	0.16
	0.09	0.11	0.03	0.1	0.13
	0.02	0.04	0.06	0.1	0.06
	0.48	0.3	0.35	0.24	0.06
	0.3	0.1	0.18	0.11	0.65
p-p38	0.70	0.60	1.64	1.34	1.17
	0.80	0.59	1.35	1.27	1.22
	0.35	0.32	0.65	0.83	0.71
	0.50	0.44	1.20	1.15	0.96
